# Mitogenomes of contemporary Spitsbergen stock bowhead whales (*Balaena mysticetus*)

**DOI:** 10.1080/23802359.2016.1258345

**Published:** 2016-11-22

**Authors:** Erik Sanderud Nyhus, Charlotte Lindqvist, Kit Kovacs, Christian Lydersen, Øystein Wiig, Lutz Bachmann

**Affiliations:** a Natural History Museum, University of Oslo, Oslo, Norway;; b Department of Biological Sciences, University at Buffalo (SUNY), Buffalo, NY, USA;; c Fram Centre, Norwegian Polar Institute, Tromsø, Norway

**Keywords:** Arctic marine mammals, baleen whales, Greenland, molecular evolution, Svalbard

## Abstract

The Spitsbergen stock of bowhead whales (*Balaena mysticetus*) is considered Critically Endangered by IUCN. Over recent decades, there have been only a few sightings, and very few biological samples are available for molecular analyses. Hence, genetic diversity of the extant Spitsbergen stock is unknown. Here, we present mitochondrial genomes from eight skin biopsy samples that were collected in 2006 and 2010. There were three different haplotypes, two of which have not previously been detected. Six samples shared the same haplotype, indicating that they were obtained from closely related whales, or possibly the same individual. Average nucleotide diversity was *π* = 0.0037, with a total of 93 variable positions among the haplotypes.

Bowhead whales (*Balaena mysticetus*) are endemic to Arctic and subarctic regions where they usually occur in close association with the sea ice. The Spitsbergen bowhead whale stock is distributed in the Greenland Sea and the northern Barents Sea. It is classified as being Critically Endangered by The International Union of Conservation of Nature (IUCN) (Reilly et al. 2012). However, the stock is thought to have been large, with an estimated 25,000–100,000 individuals, until it was driven close to extinction by extensive hunting that started in 1611 (Allen & Keay 2006). Recent suggestions for the stock size range from some few tens (Gilg & Born 2005; Wiig et al. 2007, 2010) up to approximately 100 animals (Boertmann et al. 2015), but the reality is that abundance is unknown for this population.

During ship-based surveys for bowhead whales in the Fram Strait during spring 2006, 2008, and 2010 (Wiig et al. 2007, 2010; Lydersen et al. 2012) skin biopsies were collected from eight individuals (seven in 2006 and one in 2010; Table 1). Currently, the eight skin samples represent the only contemporary biological material available for genetic studies of the Spitsbergen stock of bowhead whales. Genetic studies based on ancient bone samples from Svalbard have been reported by Borge et al. (2007).

**Table 1.** Bowhead whale samples included in this study for NGS of complete mitochondrial genomes.

Skin biopsy	Sampling locality	Collecting day	Gender^a^	Reads	coverage	Length	GenBank accession no.
A	80.58 N 2.09 E	18 April 2006	female	173,163	1515	16,390	KY026766
B	80.58 N 2.09 E	18 April 2006	female	194,888	1712	16,390	KY026767
C	80.58 N 2.09 E	18 April 2006	female	237,637	2074	16,390	KY026768
D	80.58 N 2.09 E	18 April 2006	female	238,166	2057	16,390	KY026769
E	80.58 N 2.09 E	18 April 2006	female	119,696	1033	16,390	KY026770
F	80.58 N 2.09 E	18 April 2006	female	358,755	3028	16,390	KY026771
H	81.04 N 1.16 E	26 April 2006	female	6579	117	16,389	KY026773
I	79.54 N 1.03 E	03 April 2010	female	25,197	214	16,390	KY026772
*B. mysticetus sequences from GenBank*							
	Okhotsk Sea					16,389	AP006472
	Unknown					16,390	AJ554051

^a^ Sex determination essentially according to Be´rube´ and Palsbøll (1996).

Total genomic DNA was extracted using the E.Z.N.A. Tissue DNA kit (Omega Bio-Tek) following the Tissue DNA-Spin Protocol provided with the kit. For high-throughput NGS of the genomic DNA, paired-end libraries were prepared, tagged, and analyzed on an Illumina NextSeq 500 (outsourced to StarSEQ GmbH, Mainz, Germany), and in the case of sample H in-house at the Natural History Museum Oslo on an Ion Torrent PGM^TM^ instrument.

In the present study, the complete mitochondrial genomes of the eight bowhead whale samples were assembled from quality trimmed next-generation sequencing (NGS) reads using MITObim 1.8 (Hahn et al. 2013). Six of the samples (A–F in Table 1) were collected in the same area on the same day. For all of these samples identical sequences were obtained. Accordingly, the final dataset included three different haplotypes 16,389 or 16,390 bp long. There are a total of 93 variable sites (90 transitions, 1 transversion, and 2 indels) among the contemporary Spitsbergen stock mitochondrial haplotypes. Inclusion of mitogenomes available in GenBank (AP00672 from the Okhotsk Sea and AJ554051 of unknown origin; Table 1) resulted in 115 variable sites (101 transitions, 11 transversion, and 4 indels) among the five haplotypes. Of these, 87 affect protein-coding regions (68 synonymous and 19 non-synonymous substitutions), 11 RNA genes, and 17 noncoding positions. Not surprisingly, the non-coding D-loop region was the most variable part of the mitochondrial genomes. Among the protein-coding genes, *ND4* and *ND5* showed the highest levels of positions affected by nucleotide substitutions, whereas *CoxI* appeared to be very conserved (Table 2). Average nucleotide diversity *π* was 0.0037 for the three Spitsbergen mitochondrial haplotypes, and 0.003, when the two GenBank references are included. A maximum-likelihood phylogram (Figure 1) depicts the relationships of the mitochondrial genomes with no particular grouping of the sequences obtained from the Spitsbergen stock samples.

**Figure 1.**
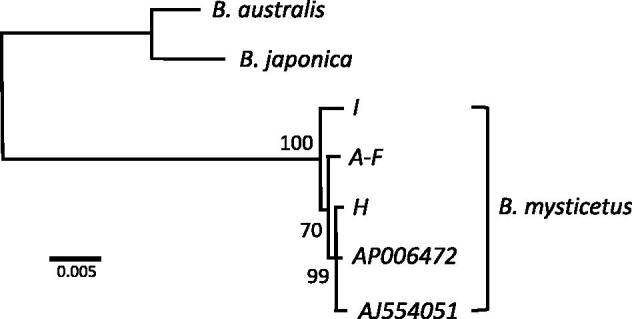
Maximum-likelihood tree based on the alignment of all available complete mitochondrial genomes of Balanenidea. Maximum-likelihood analyses were carried out in PHYML 3.0 (Guindon et al. 2010) applying the HKY85 + G + I + F model as selected by the program’s SMS option. The *Eubalaena* species *E. australis* (AP006473) and *E. japonica* (AP006474) served as an outgroup. The bootstrap support values estimated by 1000 replicates are indicated.

**Table 2.** Genetic variability of the complete bowhead whale mitochondrial genomes sequenced in this study and deposited in GenBank (accession numbers AP006472, Sasaki et al. (2005), origin: Okhotsk Sea, and AJ554051, Arnason et al. (2004), origin unknown).

Gene	% variable positions	Gene	% variable positions
*12S*	0.21	*ND3*	0.29
*16S*	0.45	*ND4L*	1.01
*ND1*	0.67	*ND4*	1.09
*ND2*	0.48	*ND5*	1.18
*CoxI*	0.13	*ND6*	0.95
*CoxII*	0.88	*CytB*	0.96
*ATP8*	0.52		
*ATP6*	0.73	*D-loop*	1.86
*CoxIII*	0.51		

Sequence comparisons of a 473 bp stretch covering the D-loop region (positions 15,461–15,934) in the alignment (supplemental material S1) with previously published sequences from bowhead whale revealed two new mitochondrial haplotypes for samples A–F and I, respectively. For sample H, the sequence of the D-loop region is identical to GenBank entry AP006472, which was assigned as haplotype F by Rooney et al. (2001), BWS1 by Borge et al. (2007), and hpdl01 by Meschersky et al. (2014). This haplotype is the most commonly detected mitochondrial haplotype among bowhead whales. The fact that the mitochondrial haplotype of samples A–F was shared in this group, and has not been detected before may indicate that it represents a rare one, and that the samples were obtained from closely related individuals. However, it cannot be excluded that they originate from the same individual as they were collected on the same day close to each other, but this can only be tested by more detailed analyses with nuclear markers.
